# HTRA1 rs11200638 variant and AMD risk from a comprehensive analysis about 15,316 subjects

**DOI:** 10.1186/s12881-020-01047-5

**Published:** 2020-05-15

**Authors:** Ying Liu, Huipeng Jin, Dong Wei, Wenxiu Li

**Affiliations:** 1 Ophthalmic function room, Hongqi Hospital Affiliated to Mudanjiang Medical College, Mudanjiang, 157000 Heilongjiang Province China; 2 Department of Ophthalmology (three disease areas), Hongqi Hospital Affiliated to Mudanjiang Medical College, Mudanjiang, 157000 Heilongjiang Province China; 3 Department of Critical Medicine, Second People’s Hospital of Mudanjiang, Mudanjiang, 157000 Heilongjiang Province China

**Keywords:** High-temperature requirement factor A1, Age-related macular degeneration, Polymorphism, Meta-analysis, Risk

## Abstract

**Background:**

The high-temperature requirement factor A1 (HTRA1) gene located at 10q26 locus has been associated with age-related macular degenerative (AMD), with the significantly related polymorphism being (rs11200638, −625G/A), however, above association is not consistent. We investigated a comprehensive analysis to evaluate the correlations between rs11200638 polymorphism and AMD susceptibility thoroughly addressing this issue.

**Methods:**

An identification was covered from the PubMed and Wanfang databases until 27th Jan, 2020. Odds ratios (OR) with 95% confidence intervals (CI) were applied to evaluate the associations. After a thorough and meticulous search, 35 different articles (33 case-control studies with HWE, 22 case-control studies about wet/dry AMD) were retrieved.

**Results:**

Individuals carrying A-allele or AA genotype may have an increased risk to be AMD disease. For example, there has a significantly increased relationship between rs11200638 polymorphism and AMD both for Asians (OR: 2.51, 95%CI: 2.22–2.83 for allelic contrast) and Caucasians [OR (95%CI) = 2.63(2.29–3.02) for allelic contrast]. Moreover, a similar trend in the source of control was detected. To classify the type of AMD, increased association was also observed in both wet (OR: 3.40, 95%CI: 2.90–3.99 for dominant model) and dry (OR: 2.08, 95%CI: 1.24–3.48 for dominant model) AMD. Finally, based on the different genotyping methods, increased relationships were identified by sequencing, TaqMan, PCR-RFLP and RT-PCR.

**Conclusions:**

Our meta-analysis demonstrated that HTRA1 rs11200638 polymorphism may be related to the AMD development, especially about individuals carrying A-allele or AA genotype, who may be as identified targets to detect and intervene in advance. Further studies using Larger sample size studies, including information about gene-environment interactions will be necessary to carry out.

## Background

In both developed and developing countries, age-related macular degeneration (AMD) is the main cause of vision loss in the elderly people [1, 2]. By 2050, about 17.8 million people will be affected by AMD [3]. AMD’s visual loss is due to dead/non-functional photoreceptor cells and potential retinal pigment epithelium cells [4]. In clinical practice, early dryness that can develop into geographic atrophy (atrophic, non-exudative) and wet (exudative) AMD characterized by choroidal neovascularization (CNV) are two forms about AMD [5, 6].

Age, race, family history, smoking and sun exposure are common risk factors [7, 8]. Another main factor in the etiology of AMD is genetic susceptibility [9]. A genome-wide association study (GWAS) in 2005 confirmed the association between AMD risk and genetic variations, suggesting that AMD is a polygenic disease [10], and in the following 15 years triggered many studies involving AMD genetic association [11–13]. So far, polymorphism about age-related maculopathy susceptibility 2 (AMRS2) rs10490924, complement factor H (CFH), complement 2 (C2)/complement factor H (CFB), complement component C3 and apolipoprotein E (APOE) haplotypes have been demonstrated as associated factors with susceptibility to AMD [14–18].

As we all known, VEGF is contributed to the progression of wet AMD, because angiogenesis and the formation of vascular permeability can lead to fluid leakage in blood vessels, and eventually lead to loss of vision [19]. Anti-VEGF drugs (eg: ranibizumab and bevacizumab) have been widely used in clinics [20, 21]. In addition, they have been shown to be effective in slowing the development of CNV, however, individual differences and shorter treatment time have been observed [22]. It is assumed that genetic factors may be involved in this period of heterogeneous response, such as variants of complement factor F (CHF), VEGFA, ARMS2 and high-temperature requirement factor A1 (HTRA1) [23–26].

HTRA1 regulates the transforming growth factor-β (TGF-β), insulin-like growth factor, and its binding protein, which is considered as regulators for cell proliferation, angiogenesis and extracellular matrix deposition. Furthermore, the inhibition of TGF-β may result in the overexpression of HTRA1 gene in wet AMD [27] (https://www.ncbi.nlm.nih.gov/gene/5654).

One of common polymorphisms in HTRA1 gene is rs11200638 (wide allele G to mutation allele A) [28]. The A-allele can influence the overexpression of HTRA1 protein, which may affect the integrity of Bruch’s membrane and lead to promote the progression of CNV stage [29].

In view of the above, we are aware of the critial role of HTRA1 gene and its common rs11200638 polymorphism, and we conducted a comprehensive summary using meta-analysis methods, including 28 different publications (33 case-control studies) [26, 30–57].

## Methods

### Search strategy and criteria

Relative studies from PubMed and Wanfang databases before 27th Jan, 2020 were searched. The keywords were “age-related macular degeneration,” “AMD,” “polymorphism or variant,” and “HTRA1 or high-temperature requirement factor A1.” Included criteria were according with as follows: (1) studies were focused on the correlation between AMD and rs11200638 polymorphism; (2) studies were all case-control and retrospective studies, and (3) the (AA, AG, and GG) genotypes both in case and control groups must be listed in Tables. Excluded criteria were consistent with as follows: (1) just case samples were shown in studies; (2) the numbers for genotypes did not shown in Tables, and (3) some duplications studies [58]. After the above conditions of the layer-by-layer screening exclusion, finally, 35 different articles were identified.

### Data extraction

Two authors (Ying Liu and Dong Wei) independently screened all papers that according using above criteria. The basic information collected from each study contains the first author, year of publication, country source for corresponding authors, race type, Hardy-Weinberg equilibrium (HWE) for control group, detecting methods and AMD disease types (dry and wet AMD) [58]..

### Statistical analysis

Odds ratios (OR) with 95% confidence intervals (CI) were applied to assess associations for rs11200638 polymorphism and AMD [58, 59]. The statistical significance of the OR was evaluated by the *Z*-test [60]. The heterogeneity among studies was calculated using the *Q*-test. Mantel-Haenszel (fixed-effects model) was selected, when *P*-value for heterogeneity is more than 0.1, otherwise, the DerSimonian-Laird (random-effects model) was applied [61, 62]. Five genetic models were adopt: AA vs. AG + GG, A vs. G, AA + AG vs. GG, AA vs. GG and AG vs. GG. The stability of results was assessed by sensitivity analysis. The HWE of control group was assessed by the Pearson’s χ^2^ test [63]. The publication bias was appraised by both Begg’s test and Egger’s test [64]. All statistical calculation were performed through Stata software (version 10.0, StataCorp LP, College Station, TX, USA) [59]. The power for sample size was calculated by Power and Sample Size Calculation Program [65].

#### Gene-gene interaction involved HTRA1 from a network

The network of gene-gene interactions for HTRA1 gene was shown by String online server to more complete understanding of the role of HTRA1 in AMD [66].

## Results

### Study searching and their basic information

**Fig. 1:**
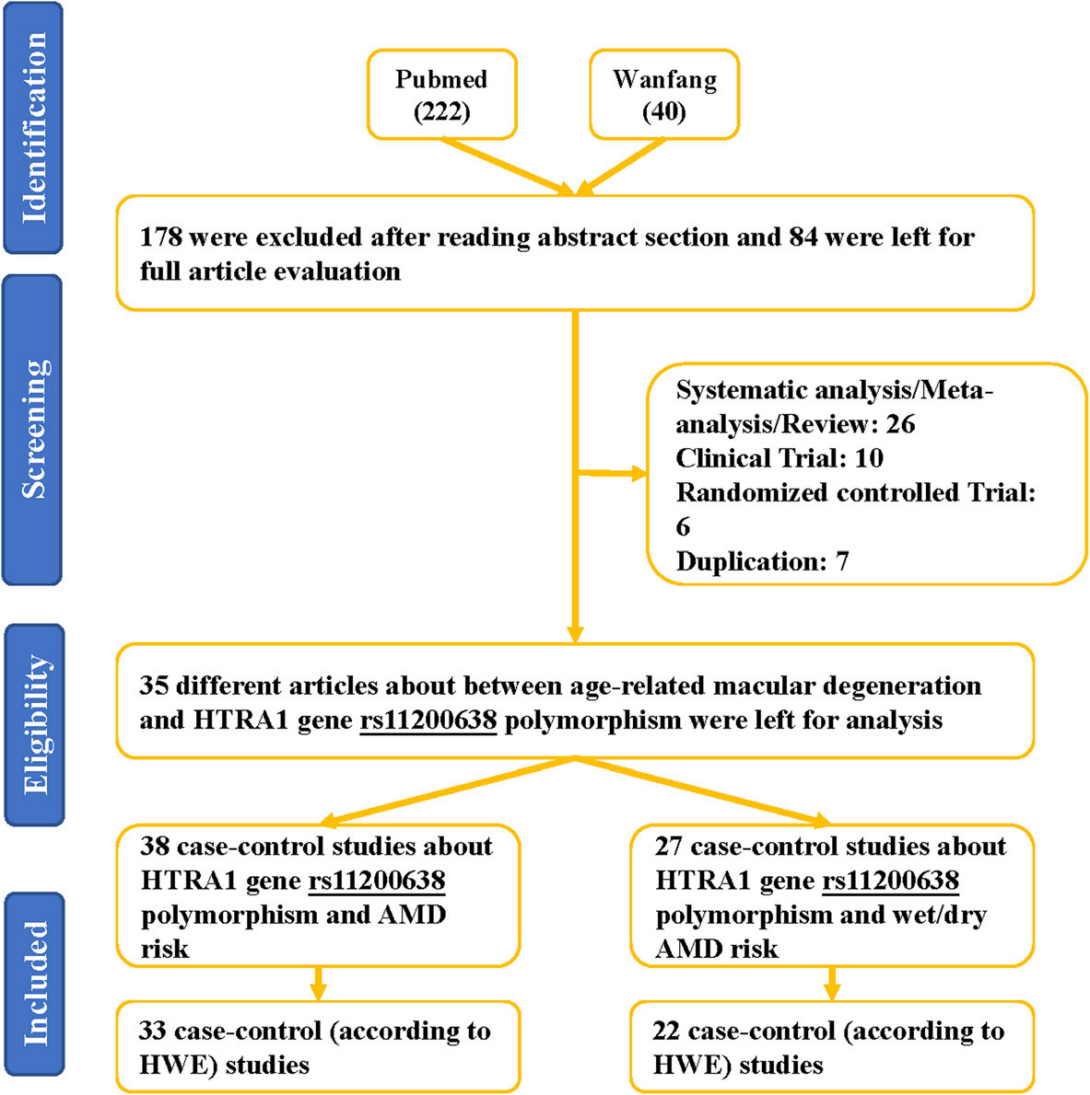
Flowchart for the search strategy was used to identify associated studies for rs11200638 polymorphism and AMD risk

**Fig. 2:**
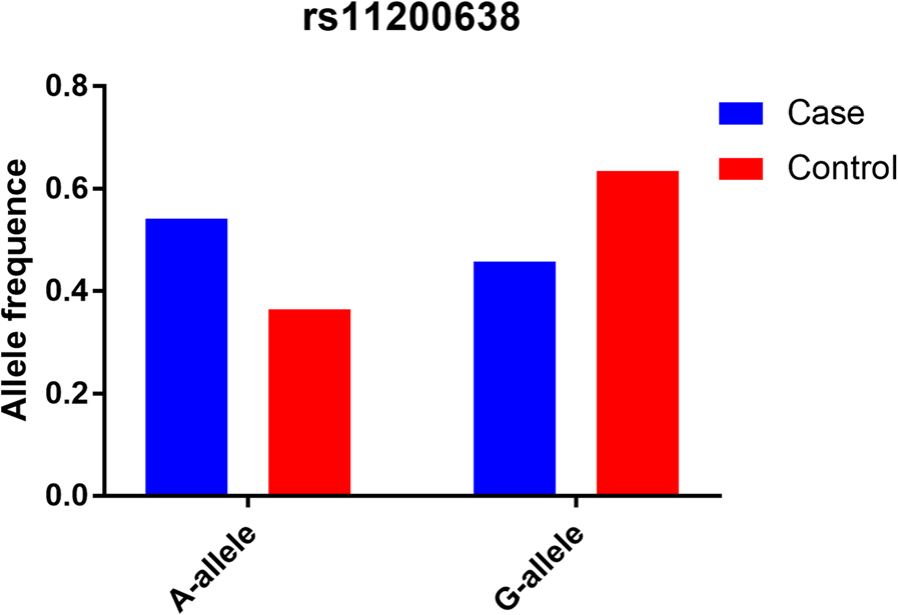
The MAF of minor-allele (mutant-allele) for HTRA1 gene rs11200638 polymorphism from the 1000 Genomes online database and present analysis. EAS: East Asian; EUR: European; AFR: African; AMR: American; SAS: South Asian

**Fig. 3:**
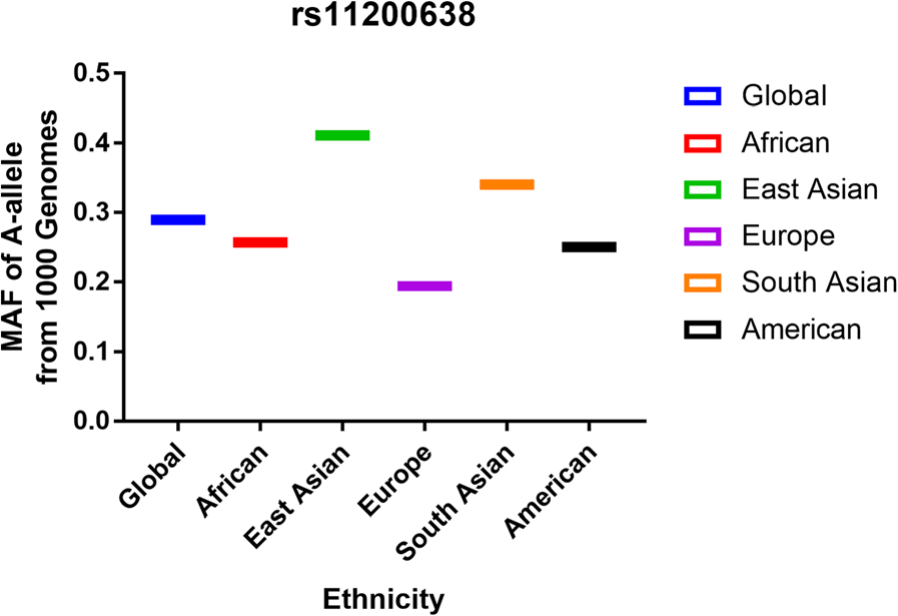
Frequency about A-allele for the rs11200638 polymorphism among cases or controls by ethnicity

A number of 262 papers were garnered by a document from the PubMed (222 titles) and Wanfang (40 titles) databases. One hundred seventy-eight articles were deleted after reading over the abstract sections (Fig. 1). Forty-one articles were excluded due to duplication (7), meta-analysis or systematic analysis (26), clinical trial (10), randomized controlled trial (6). Finally, 35 different articles [26, 30–57, 67–71] were selected, including 38 studies about HTRA1 gene rs11200638 polymorphism and AMD risk (Table 1) and 27 case-control studies about HTRA1 gene rs11200638 polymorphism and wet or dry AMD risk (Table 2). Five case-control studies [67–71] were not consistent with HWE in control groups. To make our analysis to more strict, we deleted above five studies, so there were about 33 case-control studies (8101 cases and 7215 controls) for the whole AMD [26, 30–57], and 22 case-control studies for wet or dry (3938 cases and 4427 controls) studies [26, 30, 31, 33, 34, 40, 41, 43, 44, 46–48, 51, 53–56]. The A-allele frequency from case group was observed as higher in control individuals (54.2% vs. 36.5%) (Fig. 2, Supplementary Table 1). Nineteen studies were Asian samples, and 14 from Caucasian population; source of control in 22 studies were hospital-based (HB), and 11 were from population-based (PB); 17 case-control studies were about wet AMD disease, and 5 were about dry disease. Additional, the Minor Allele Frequency (MAF) about five main worldwide populations in the 1000 Genomes Browser was shown in Fig. 3: Global (0.290); East Asian (EAS = 0.411); European (EUR = 0.194); African (AFR = 0.257); American (AMR = 0.250); and South Asian (SAS = 0.340) (Supplementary Table 1).

**Table 1:** Characteristics of included studies in HTRA1 rs11200638 polymorphism and AMD risk

Author	Year	Country	Ethnicity	type	Case	Control	SOC	Cases			Controls			HWE	Genotype
								**AA**	**AG**	**GG**	**AA**	**AG**	**GG**		
Tian	2012	China	Asian	AMD	532	468	HB	255	193	84	104	224	140	0.423	Typer 4.0 software
Ruamviboonsuk	2017	Thailand	Asian	wet	377	1073	PB	125	164	88	146	490	437	0.643	InfiniumOmniExpressExome-8 v1.3 platform
Chu	2008	China	Asian	wet	144	126	HB	76	52	16	31	69	26	0.276	PCR-RFLP
Losonczy	2011	Hungary	Caucasian	AMD	103	95	HB	23	50	30	3	49	43	0.133	PCR-RFLP
Tuo	2008	USA	Caucasian	AMD	142	132	PB	33	60	49	7	51	74	0.638	PCR-RFLP
Tuo	2008	USA	Caucasian	AMD	330	191	PB	63	164	103	12	73	106	0.904	PCR-RFLP
Tuo	2008	USA	Caucasian	AMD	272	555	PB	15	122	135	20	186	349	0.431	PCR-RFLP
Tuo	2008	USA	Caucasian	AMD	46	22	PB	2	15	29	1	6	15	0.696	PCR-RFLP
Chan	2007	USA	Caucasian	AMD	52	13	HB	14	27	11	0	8	5	0.109	RT-PCR
Kanda	2007	USA	Caucasian	AMD	457	280	HB	102	183	172	11	90	179	0.94	RT-PCR
Cheng	2013	China	Asian	wet	93	93	HB	52	30	11	21	42	30	0.395	Sequencing
Liang	2012	China	Asian	wet	161	150	HB	61	83	17	30	72	48	0.751	Sequencing
Jiang	2008	China	Asian	wet	159	140	HB	99	47	13	31	67	42	0.662	Sequencing
Lee	2010	Korea	Asian	wet	137	187	HB	57	59	21	35	100	52	0.283	Sequencing
Lin	2008	China-Taiwan	Asian	AMD	95	90	HB	53	33	9	19	47	24	0.651	Sequencing
Kaur	2008	India	Asian	AMD	229	184	HB	90	89	50	21	85	78	0.765	Sequencing
Xu	2007	China	Asian	wet	121	132	HB	56	52	13	24	64	44	0.931	Sequencing
Tam	2008	China-Hong Kong	Asian	wet	163	183	HB	94	51	18	38	90	55	0.916	Sequencing
Mori	2007	Japan	Asian	AMD	123	133	HB	45	52	26	22	57	54	0.298	Sequencing
Askari	2015	Iran	Asian	AMD	120	120	HB	58	42	20	12	66	42	0.057	Sequencing
Lana	2018	Brazil	Caucasian	AMD	204	166	HB	73	89	42	22	77	67	0.987	Sequencing
Kaur	2013	India	Asian	AMD	198	145	PB	84	70	44	17	67	61	0.829	Sequencing
Ng	2016	Hong Kong	Asian	wet	194	183	PB	109	63	22	38	90	55	0.916	Sequencing
Kaur	2013	India	Caucasian	AMD	616	426	PB	130	292	194	17	138	271	0.913	Sequencing
Chen	2013	China	Asian	AMD	158	157	HB	28	74	56	21	77	59	0.599	TaqMan
Kondo	2007	Japan	Asian	AMD	73	94	HB	29	39	5	16	40	38	0.334	TaqMan
Leveziel	2007	France	Caucasian	wet	118	116	HB	32	57	29	5	41	70	0.743	TaqMan
Weger	2007	Austria	Caucasian	wet	242	157	PB	67	108	67	8	50	99	0.609	TaqMan
Lu	2007	China	Asian	wet	90	106	HB	53	34	3	15	63	28	< 0.05	PCR-RFLP
Cruz-González	2013	Spain	Caucasian	AMD	121	91	HB	29	60	32	61	21	9	< 0.05	PCR-RFLP
Mohamad	2019	Malaysia	Asian	wet	145	145	HB	79	47	19	48	82	15	< 0.05	PCR-RFLP
Yang	2010	China	Asian	wet	109	150	HB	31	45	33	30	50	70	< 0.05	PCR-RFLP
Chen	2008	USA	Caucasian	AMD	776	294	HB	131	400	245	10	128	156	< 0.05	Sequencing
Zeng	2011	China	Caucasian	AMD	1335	509	PB	244	641	450	21	181	307	0.374	SNaPshot
Li	2015	China	Asian	AMD	146	145	HB	73	54	19	44	74	27	0.674	MassARRAY MALDI-TOF
Yang	2018	China	Asian	AMD	201	201	HB	103	74	24	42	100	59	0.975	MassARRAY MALDI-TOF
Matuskova	2020	Czech Republic	Caucasian	wet	307	191	HB	69	148	90	9	66	116	0.921	SNaPshot Multiplex-System
Fritsche	2008	Germany	Caucasian	AMD	760	549	PB	152	344	264	22	174	353	0.923	Multiplex PCR

*HB* hospital-based; *PB* population-based; *SOC* source of control; *PCR-RFLP* polymerase chain reaction followed by restriction fragment length polymorphism; *RT-PCR* real-time PCR; *MALDI-TOF* polymerase chain reaction–matrix-assisted laser desorption/ionization time-of-flight; *HWE* Hardy–Weinberg equilibrium of control group

**Table 2:** Characteristics of included studies in HTRA1 rs11200638 polymorphism and wet/dry AMD risk, respectively

Author	Year	Country	Ethnicity	type	Case	Control	SOC	Cases			Controls			HWE	Genotype
								AA	AG	GG	AA	AG	GG		
Lin	2008	China-Taiwan	Asian	dry	52	90	HB	28	19	5	19	47	24	0.651	Sequencing
Chan	2007	USA	Caucasian	dry	18	13	HB	4	10	4	0	8	5	0.109	RT-PCR
Mori	2007	Japan	Asian	dry	19	116	HB	4	7	8	5	41	70	0.743	Sequencing
Askari	2015	Iran	Asian	dry	32	120	HB	11	12	9	12	66	42	0.576	Sequencing
Ruamviboonsuk	2017	Thailand	Asian	wet	377	1073	PB	125	164	88	146	490	437	0.643	InfiniumOmniExpressExome-8 v1.3 platform
Cheng	2013	China	Asian	wet	93	93	HB	52	30	11	21	42	30	0.395	Sequencing
Ng	2016	Hong Kong	Asian	wet	194	183	PB	109	63	22	38	90	55	0.915	Sequencing
Liang	2012	China	Asian	wet	161	150	HB	61	83	17	30	72	48	0.75	Sequencing
Chu	2008	China	Asian	wet	144	126	HB	76	52	16	31	69	26	0.276	PCR-RFLP
Jiang	2008	China	Asian	wet	159	140	HB	99	47	13	31	67	42	0.662	Sequencing
Lee	2010	Korea	Asian	wet	137	187	HB	57	59	21	35	100	52	0.283	Sequencing
Lin	2008	China-Taiwan	Asian	wet	43	90	HB	25	14	4	19	47	24	0.651	Sequencing
Xu	2007	China	Asian	wet	121	132	HB	56	52	13	24	64	44	0.931	Sequencing
Tam	2008	China-Hong Kong	Asian	wet	163	183	HB	94	51	18	38	90	55	0.916	Sequencing
Chan	2007	USA	Caucasian	wet	31	13	HB	8	16	7	0	8	5	0.109	RT-PCR
Leveziel	2007	France	Caucasian	wet	118	116	HB	32	57	29	5	41	70	0.743	TaqMan
Mori	2007	Japan	Asian	wet	104	116	HB	41	45	18	5	41	70	0.743	Sequencing
Askari	2015	Iran	Asian	wet	88	120	HB	47	30	11	12	66	42	0.576	Sequencing
Weger	2007	Austria	Caucasian	wet	242	157	PB	67	108	67	8	50	99	0.609	TaqMan
Lu	2007	China	Asian	wet	90	106	HB	53	34	3	15	63	28	< 0.05	PCR-RFLP
Mohamad	2019	Malaysia	Asian	wet	145	145	HB	79	47	19	48	82	15	< 0.05	PCR-RFLP
Yang	2010	China	Asian	wet	109	150	HB	31	45	33	30	50	70	< 0.05	PCR-RFLP
Chen	2008	USA	Caucasian	wet	470	294	HB	76	245	149	10	128	156	< 0.05	Sequencing
Chen	2008	USA	Caucasian	dry	306	294	HB	55	155	96	10	128	156	< 0.05	Sequencing
Zeng	2011	China	Caucasian	dry	341	509	PB	60	166	115	21	181	307	0.374	SNaPshot
Zeng	2011	China	Caucasian	wet	994	509	PB	184	475	335	21	181	307	0.374	SNaPshot
Matuskova	2020	Czech Republic	Caucasian	wet	307	191	HB	69	148	90	9	66	116	0.921	SNaPshot Multiplex-System

### Quantitative synthesis

#### Total analysis

**Fig. 4:**
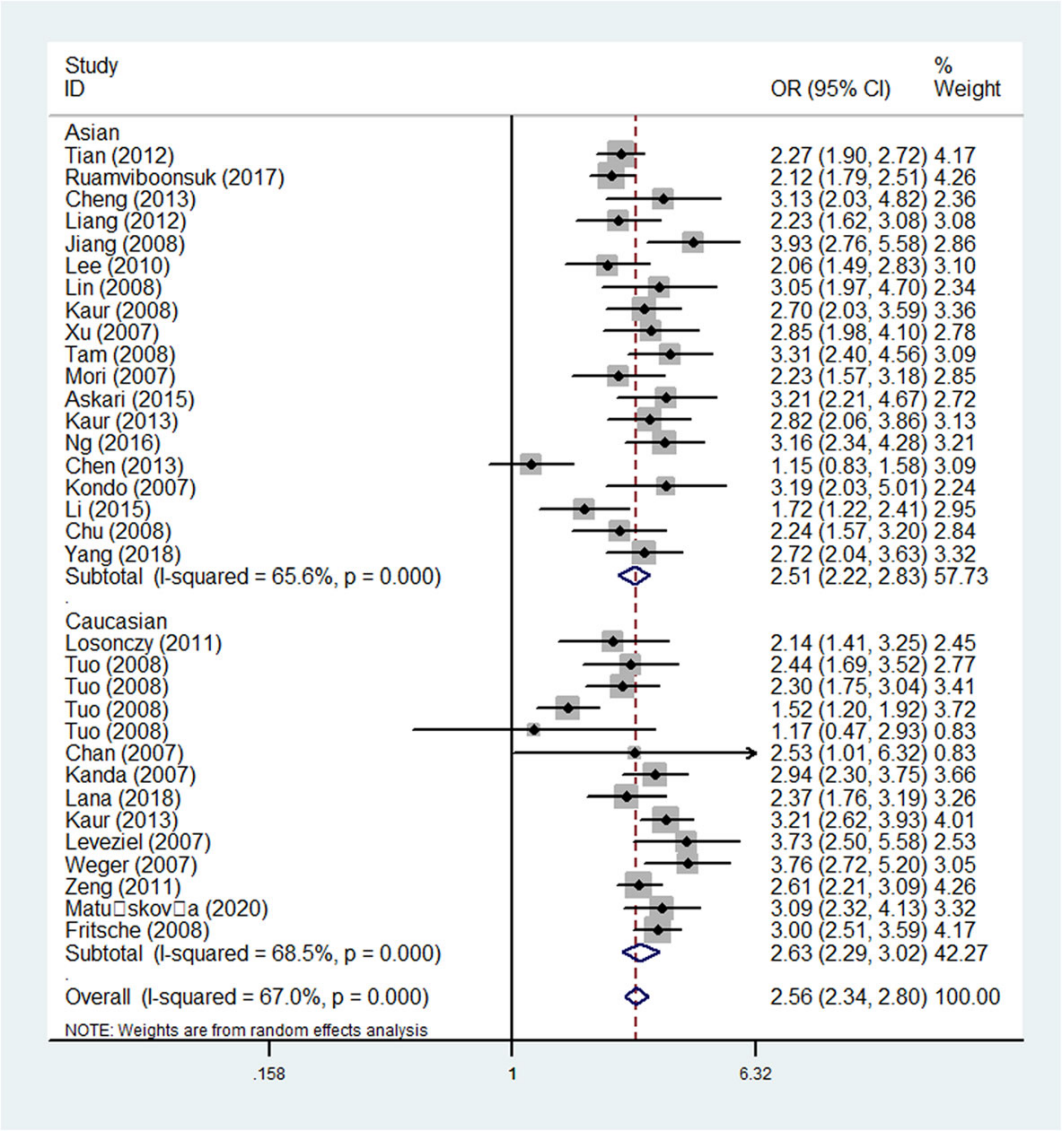
Forest plot of AMD risk associated with HTRA1 gene rs11200638 polymorphism (A-allele vs. G-allele) by ethnicity subgroup

Increasing relationships were found for rs11200638 and AMD risk in all models (eg: AA vs. GG: OR = 5.45, 95CI% = 4.26–6.98, *P* < 0.001) (Table 3). In order to make this study more convincing and reliable, we detected five studies, which were not according with HWE, finally, we tested the 33 case-control studies. Also significantly increasing correlations were observed in whole models [for example: allelic contrast: OR (95%CI): 2.56(2.34–2.80), *P* < 0.001; AA+AG vs. GG: OR (95%CI): 2.80 (2.49–3.15), *P* < 0.001] (Fig. 4) (Table 3).

**Table 3:** Results of the meta-analysis on HTRA1 rs11200638 polymorphism and AMD risk in total and types of subgroups

Variables	N	Case/	A-allele vs. G-allele	AG vs. GG	AA+AG vs. GG	AA vs. GG	AA vs. AG + GG
		Control	OR(95%CI) *P*_h_*P*	OR(95%CI) *P*_h_*P*	OR(95%CI) *P*_h_*P*	OR(95%CI) *P*_h_*P*	OR(95%CI) *P*_h_*P*
Total	38	8582/7452	2.39(2.12–2.69)0.000 0.000	1.91(1.69–2.16)0.000 0.000	2.63(2.30–3.01)0.000 0.000	5.45(4.26–6.98)0.000 0.000	3.75(3.04–4.63)0.000 0.000
HWE	33	8101/7215	2.56(2.34–2.80)0.000 0.000	1.98(1.76–2.23)0.001 0.000	2.80(2.49–3.15)0.000 0.000	6.13(5.09–7.38)0.000 0.000	4.10(3.55–4.73)0.000 0.000
Ethnicity							
Asian	19	3424/4004	2.51(2.22–2.83)0.000 0.000	1.67(1.47–1.88)0.167 0.000	2.68(2.25–3.19)0.009 0.000	5.36(4.31–6.66)0.003 0.000	3.70(3.16–4.35)0.009 0.000
Caucasian	14	4677/3211	2.63(2.29–3.02)0.000 0.000	2.37(2.15–2.61)0.140 0.000	2.94(2.51–3.45)0.004 0.000	7.52(5.62–10.07)0.014 0.000	5.00 (3.91–6.40)0.070 0.000
SOC							
HB	22	3589/3273	2.56(2.28–2.88)0.000 0.000	1.86(1.58–2.19)0.025 0.000	2.81(2.38–3.31)0.005 0.000	5.99(4.74–7.59)0.001 0.000	3.93(3.31–4.68)0.005 0.000
PB	11	4512/33942	2.55(2.18–2.99)0.000 0.000	2.16(1.84–2.53)0.021 0.000	2.80(2.35–3.35)0.001 0.000	6.37(4.64–8.76)0.001 0.000	4.43(3.41–5.75)0.009 0.000
AMD type							
Wet	17	3476/3579	3.03(2.59–3.55)0.000 0.000	2.11(1.81–2.46)0.138 0.000	3.40(2.90–3.99)0.073 0.000	7.65(5.73–10.21)0.009 0.000	4.65(3.72–5.82)0.008 0.000
Dry	5	462/848	2.36(1.71–3.24)0.750 0.000	1.33(0.76–2.32)0.705 0.316	2.08(1.24–3.48)0.618 0.005	6.01(3.05–11.87)0.889 0.000	4.77(2.79–8.16)0.964 0.000
Genotyping							
Others	5	3004/2599	2.55(2.22–2.94)0.027 0.000	2.14(1.67–2.73)0.006 0.000	2.85(2.38–3.43)0.055 0.000	6.25(4.26–9.15)0.005 0.000	4.18 (3.14–5.57)0.033 0.000
Sequencing	14	2613/2332	2.84(2.61–3.09)0.237 0.000	2.08(1.81–2.41)0.252 0.000	3.19(2.79–3.65)0.710 0.000	7.00(5.84–8.39)0.677 0.000	4.51(3.90–5.21)0.169 0.000
TaqMan	4	591/524	2.66(1.43–4.94)0.000 0.002	2.79(1.31–5.91)0.000 0.008	3.61(1.52–8.60)0.000 0.004	7.52(2.05–27.68)0.000 0.002	3.86(1.66–8.98)0.002 0.002
PCR-RFLP	6	1037/1121	1.98(1.72–2.26)0.105 0.000	1.76(1.45–2.14)0.611 0.000	2.08(1.73–2.50)0.411 0.000	4.30(2.51–7.35)0.073 0.000	3.33(2.09–5.31)0.095 0.000
RT-PCR	2	509/293	2.91(2.30–3.69)0.755 0.000	2.08(1.51–2.86)0.643 0.000	2.90(2.15–3.92)0.734 0.000	9.83(5.18–18.65)0.817 0.000	7.19(3.84–13.45)0.806 0.000
MassARRAY MALDI-TOF	2	347/346	2.18(1.39–3.49)0.043 0.001	1.46(0.95–2.24)0.213 0.088	2.22(1.13–4.38)0.099 0.021	3.84(1.53–9.63)0.044 0.004	3.05(1.78–5.23)0.097 0.000

*P*_h_: value of *Q*-test for heterogeneity test; *P*: *Z*-test for the statistical significance of the OR

#### Subgroup analysis

**Fig. 5:**
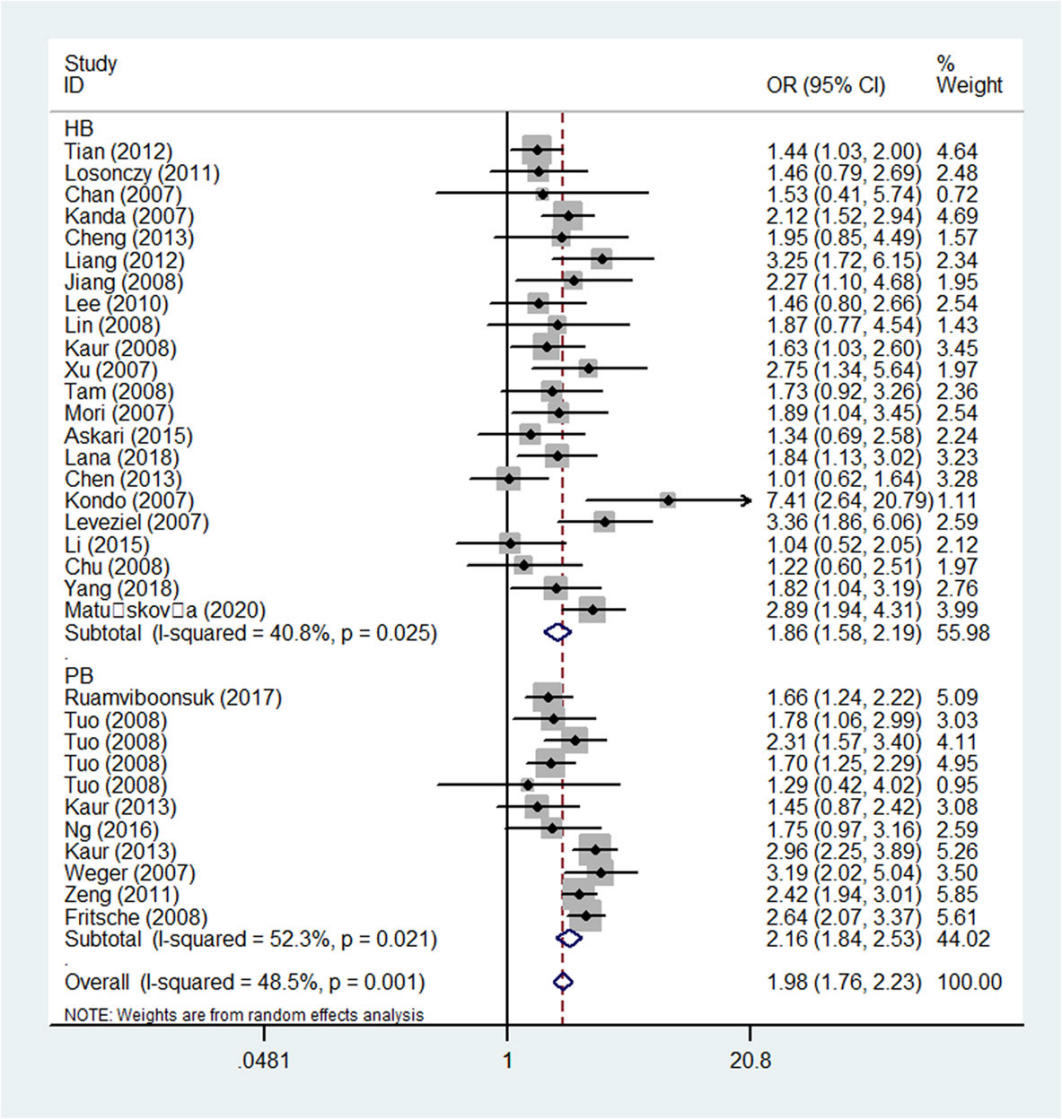
Forest plot of AMD risk associated with HTRA1 gene rs11200638 polymorphism (AG vs. GG) by source of control subgroup

**Fig. 6:**
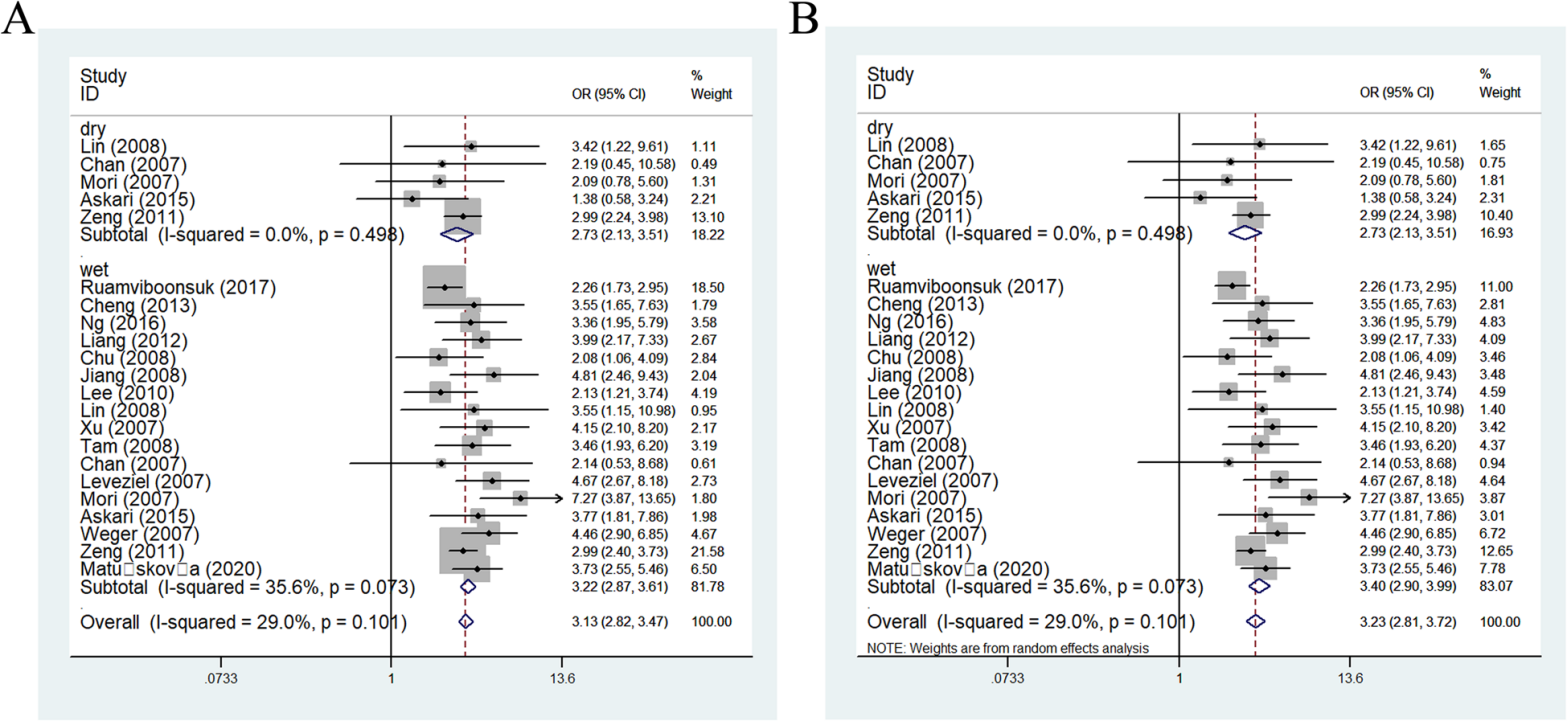
Forest plot of AMD risk associated with HTRA1 gene rs11200638 polymorphism (AA+AG vs. GG) by AMD type subgroup. **a**: wet AMD; **b**: dry AMD

**Fig. 7:**
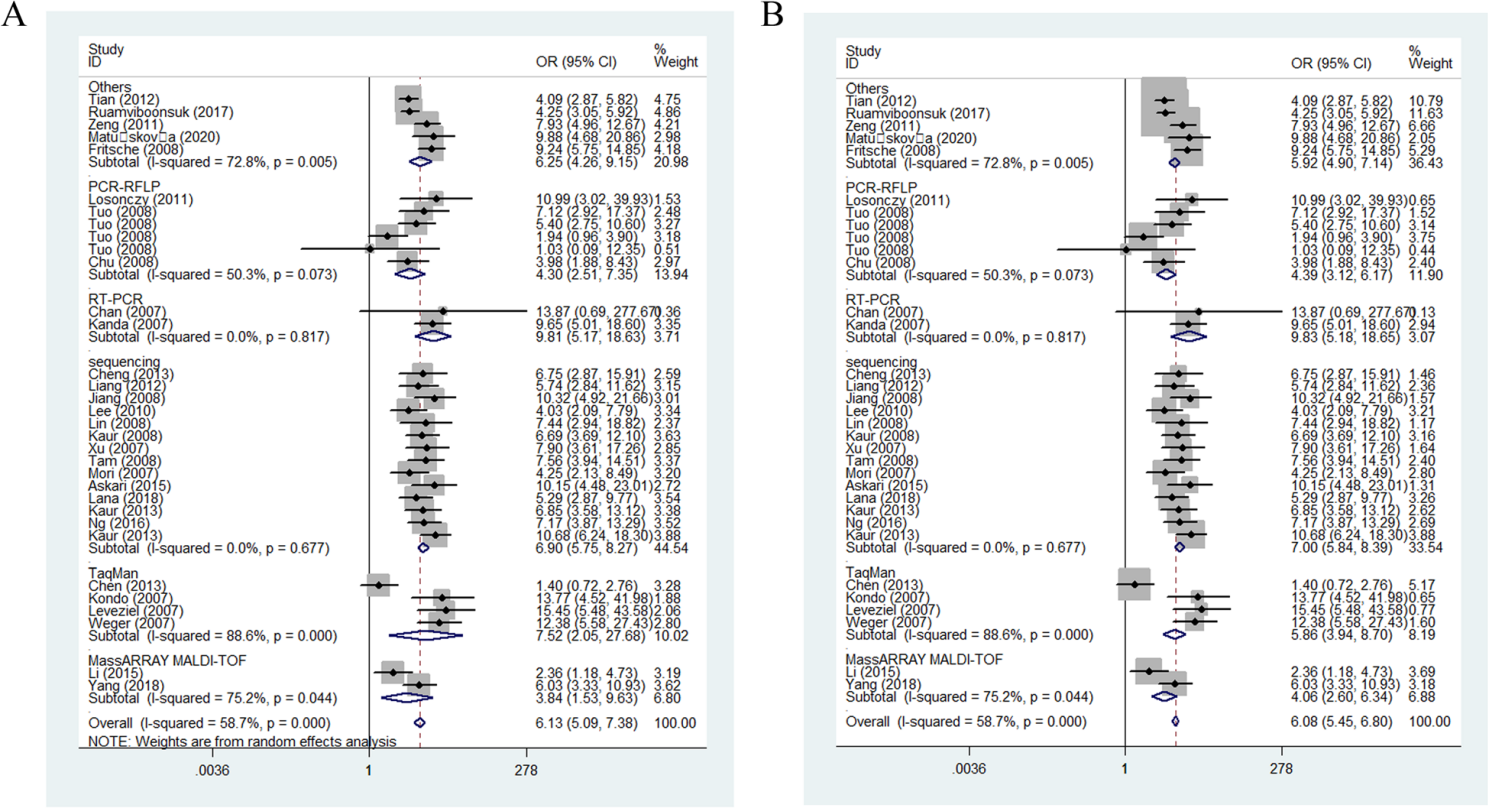
Forest plot of AMD risk associated with HTRA1 gene rs11200638 polymorphism (AA vs. GG) by genotyping methods subgroup. **a**: random model; **b**: fixed model

Coming up, we all know that the frequency of A-allele in different races was not the same, so we tried to analysis the relationships by ethnicity subgroups in further, which indicated an incremental statistically association between this polymorphism and both in Asians [OR(95% CI) = 2.51(2.22–2.83), *P*_*(*heterogeneity)_ < 0.001, *P* < 0.001 in allelic contrast, Fig. 4; AA vs. AG + GG: OR (95% CI) = 3.70(3.16–4.35), *P*_*(*heterogeneity)_ = 0.009, *P* < 0.001] and Caucasian populations [OR (95% CI) = 2.94(2.51–3.45), *P*_*(*heterogeneity)_ < 0.001 in dominant model; OR = 2.37, 95% CI = 2.15–2.61, *P*_*(*heterogeneity)_ < 0.001 in heterozygote comparison; allelic comparison, OR = 2.63, 95% CI = 2.29–3.02, *P*_heterogeneity_ < 0.001, *P* < 0.001, Fig. 4) (Table 3). In addition, regular analysis by source of control, also similar results were detected in both PB and HB studies [AG vs. GG: OR (95% CI) = 1.86(1.58–2.19), *P*_heterogeneity_ = 0.025, *P* < 0.001 in HB; AG vs. GG: OR (95% CI) = 2.16(1.84–2.53), *P*_*(*heterogeneity)_ = 0.021, *P* < 0.001 in PB] (Table 3) (Fig. 5). AMD have different types and stages, the different of clinical presentation for dry and wet AMD is completely different, so we firmly believed that the correlations existed should be evaluated separately, significant positive associations were found both for dry (eg. AA+AG vs. GG: OR (95% CI) = 2.73(2.13–3.51), *P*_*(*heterogeneity)_ = 0.498, *P* < 0.001, Fig. 6a) and wet AMD (for example in AA+AG vs. GG model: OR = 3.40, 95% CI = 2.90–3.99, *P*_heterogeneity_ = 0.073, *P* < 0.001, Fig. 6b). Finally, we tried to in each method, whether associations may exist in our analysis, we found some positive results in some methods (such as in AA vs. GG model: OR = 7.52, 95% CI = 2.05–27.68, *P*_heterogeneity_ < 0.001, *P* = 0.002 about TaqMan; OR = 4.30, 95% CI = 2.51–7.35, *P*_heterogeneity_ = 0.073 about PCR-RFLP, OR = 3.84, 95% CI = 1.53–9.63, *P*_heterogeneity_ = 0.044 about MassARRAY MALDI-TOF, Fig. 7a; OR = 7.00, 95% CI = 5.84–8.39, *P*_heterogeneity_ = 0.677 about sequencing, OR = 9.83, 95% CI = 5.18–18.65, *P*_heterogeneity_ = 0.817 about sequencing RT-PCR, Fig. 7b) (Table 3).

### Bias diagnosis for publication and sensitivity analysis

Begg’s funnel plot and Egger’s test were applied to assess publication bias. At beginning, the funnel plots seemed asymmetrical in allele comparison for rs11200638 by Begg’s test, suggesting no publication bias was existed. Then, Egger’s test was applied to assess the funnel plot symmetry. As a result, no publication bias was observed [eg. allelic contrast, Egger’s test (*t* = 0.89, *P* = 0.38); Begg’s test (*z* = 0.85, *P* = 0.396), Supplementary Figure 1A,B] (Table 4).

**Table 4:** Publication bias tests (Begg’s funnel plot and Egger’s test for publication bias test) for HTRA1 rs11200638 polymorphism

Egger’s test						Begg’s test	
Genetic type	Coefficien*t*	Standard error	*t*	*P* value	95%CI of intercept	*z*	*P* value
A-allele vs. G-allele	0.211	0.924	0.23	0.820	(−1.673–2.096)	0.42	0.676
AG vs. GG	−0.031	0.500	−0.06	0.951	(−1.051–0.989)	0.29	0.768
AA+AG vs. GG	−0.045	0.532	−0.08	0.933	(−1.130–1.040)	0.26	0.792
AA vs. GG	0.297	0.382	0.78	0.441	(−0.481–1.076)	0.36	0.722
AA vs. AG + GG	0.365	0.438	0.83	0.412	(−0.529–1.258)	0.60	0.546

To assess the power and stability of whole study and each study, the sensitive analysis was adopt to carry out, as a result, no significant showing were found (Supplementary Figure 2).

### Gene-gene network from string online site

**Fig. 8:**
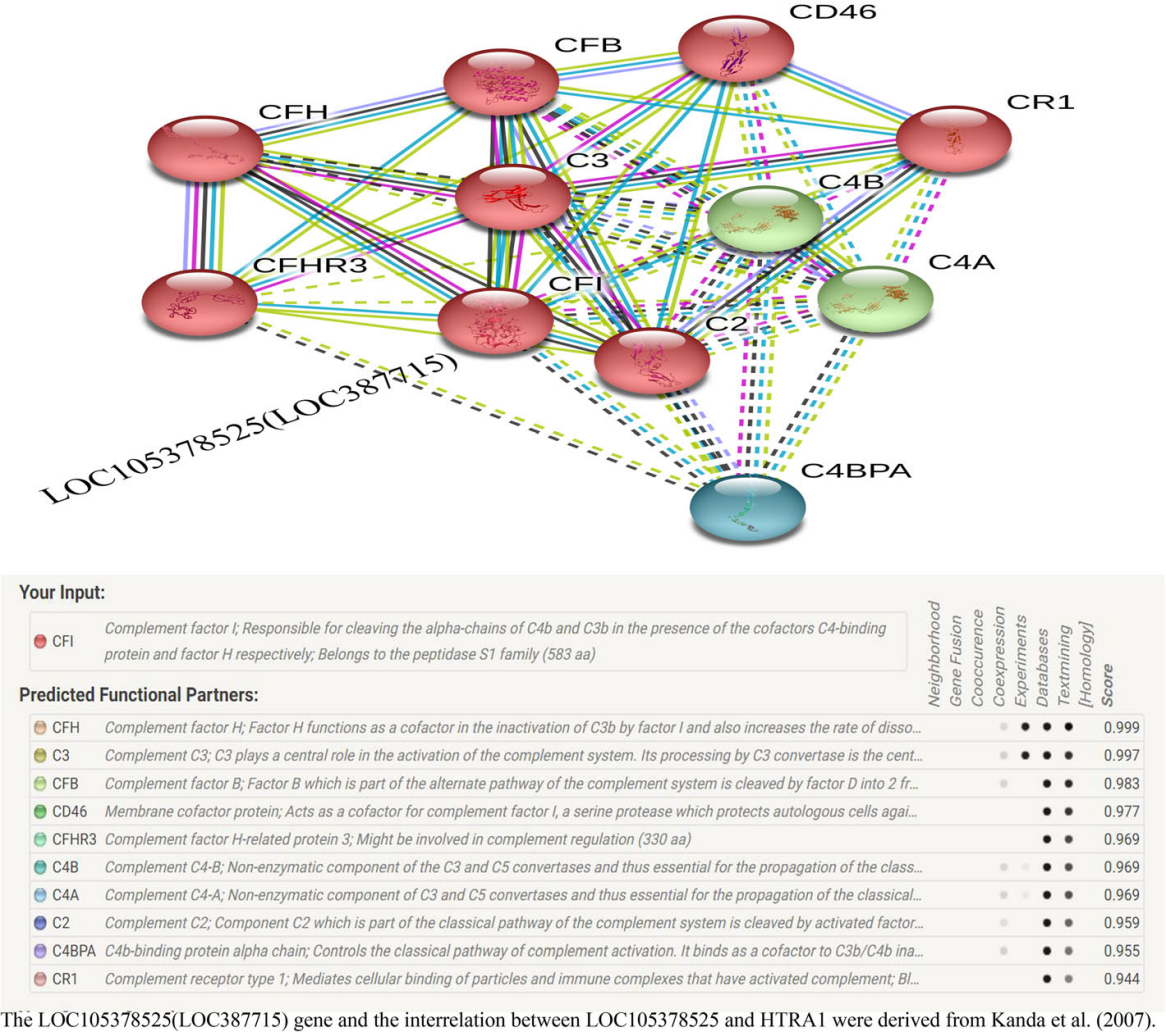
Interactions network about human HTRA1 gene and other related genes from String server. ACAN: aggrecan core protein; ARMS2: age-related maculopathy susceptibility; CLPP: ATP-dependent Clp protease proteolytic subunit; CTRC: chymotrypsin-C; YME1L1: ATP-dependent zine metalloprotease YME1L1; CFH: complement factor H; HSPD1: 60 kDa heat shock protein; RPL34: 60S ribosomal protein L34; CLPX: ATP-dependent Clp protease ATP-binding subunit clpX-like; PLEKHG4: Puratrophin-1

The HTRA1 gene may interacts with numerous genes from String online server (Fig. 8).

## Discussion

Due to the severe consequences of vision loss caused by AMD, especially advanced AMD (atrophic/dry or neovascular/wet), it is necessary to study its etiology and mechanism, and then develop early diagnostic methods and effective treatments. Today, VEGF inhibitors have been widely regarded as effective drugs in clinical application for CNV (wet AMD) [3, 72, 73]. Therefore, identifying some novel detection markers and target drugs for some different types of AMD is the focus of current and future research. In the introduction, we clarified that genetic factors may help us to search for AMD in potential high-risk groups, which can be prevented and treated in advance.

In our analysis, we selected the HTRA1 gene that can regulate certain growth factors. The rs11200638 polymorphism in HTRA1 is the most common single nucleotide polymorphism (SNP) and has been received attention. However, Kanda et al. [35] demonstrated that there was no HTRA1 gene involved in AMD related SNPs, and its rs11200638 polymorphism did not appear to have an effect on the transcripts. Instead, they found a putative mitochondrial protein (LOC105378525) that may be expressed in the retina in the negative strand, which may be a candidate gene. In fact, they showed that rs11200638 and rs10490924 are in a strong linkage disequilibrium, which is a predicted non-synonymous A69S change in a protein named LOC105378525(LOC387715)/ARMS2. According to their research, rs10490924 was a strong candidate SNP associated with AMD risk, not rs11200638. In addition, Bonyadi et al. [74] conducted a meta-analysis of rs10490924 and found that the combined cigarette smoking and rs10490924 polymorphism may have significant association with AMD risk. We believed rs10490924 was a valuable SNP for AMD, nevertheless, conclusions based on a single study may not be negated by the potential functions for HTRA1 and its SNPs, which need more evidences and support from published and future researches.

Mori et al. before all others reported the correlations for rs11200638 polymorphism and AMD [47]. After that, researchers imitated similar works involving different ethnicities and different types about AMD. Nevertheless, each conclusion was indecipherable, even same population, though two published meta-analysis. As we all know, meta-analysis offers a method combining all related studies to acquire a powerful genetic effects for disease susceptibility [75].

Two previous meta-analysis [76, 77] about rs11200638 polymorphism and AMD have been reported, however, each study has its limitations. For example, Tang et al. just included fourteen case-control studies, two studies [67, 69] were not consistent with HWE, and Tuo et al. actually reported four-source case-control studies, which shouldn’t be combined together [77]. Chen et al. also performed a same study in the same year including 14 case-control studies, similar limitations were existed [76]. After year of 2008, newly added studies have been published, and to perfect the above deficiencies, we carried out a comprehensive analysis to come to a more convincing conclusion about HTRA1 gene rs11200638 polymorphism and AMD susceptibility.

Our current research is the comprehensive analysis about the associations between HTRA1 gene rs11200638 polymorphism and AMD, involving 8101 AMD samples and 7215 controls. Increased associations were found in the whole group, in Asian and Caucasian subgroups, source of control subgroup, and dry/wet sub-types of AMD, different genotyping methods (Sequencing, TaqMan, PCR-RFLP, RT-PCR and MassARRAY MALDI-TOF), which means that A-allele or AA genotype is the risk factor for AMD, in other words, if individuals carry on this SNP from peripheral blood test, which may indicate that it is possible to increase the occurrence of AMD for them in present time or at some point in the future. Therefore, this polymorphism may be helpful in screening vulnerable populations for AMD in advance. In addition, the power of present study was 1.00, which suggested our conclusions were stable and convincing.

In addition, the online String website was used to make a forecast several potential and functional genes associated with HTRA1. As a result, ten genes were predicted. Among them, the highest score of association was ACAN (0.943), however, so far, no research has been reported between this gene and AMD and interaction between this gene and HTRA1. Future research should be payed attention to above information, which may be in favor of AMD early detection/prevention and intervention. In other partners, ARMS2 and CFH have been shown to associate with AMD. Both *ARMS2* and *HTRA1* genes have a linkage disequilibrium, which is located nearby form 10q26 chromosome. ARMS2 rs10490924 was related to response to ranibizumab treatment among wet AMD patients [70]. CFH gene T1277C polymorphism is strong associated with both wet and dry AMD and may be contribute to the inflammation in the pathogenesis of AMD [78]. As for the rest interaction genes (CLPP, CTRC, YME1L1, HSPD1, RPL34, CLPX and PLEKHG4) both had moderate score and no literature to support. It seems that above ten genes associated with HTRA1 came from text mining scores, which were derived from the co-occurrence of gene/protein names in related abstracts. In addition, it was important considered the occurrence of the LOC105378525 (LOC387715) and its polymorphism (A69S, rs10490924) as the main factor for AMD reported by Kanda et al. (2007) [35], which should be added in the network of HTRA1 related genes. In a word, we should deep explore these partners of HTRA1 gene, and gene-gene interactions in the development of AMD in the next step.

Some limitations should be declared. First of all, Mixed and African individuals should be paid more attention in future studies, which was vacant in present analysis. Second, analysis about gene-gene/gene-environment interactions should be added, because some specific environmental and lifestyle factors may influence associations between rs11200638 polymorphism and AMD (such as hypertension, familial history, age range, diabetes stage, smoking status). Third, vision is the most concerned-clinical indicator of AMD, future studies should include the value of the vision and analyze the relationships between rs11200638 polymorphism and the degree of visual impairment, which may help us to better detect disease progression.

## Conclusion

In conclusion, our present analysis demonstrated HTRA1 rs11200638 polymorphism may play a risk factor for the susceptibility of AMD, larger and more comprehensive studies should be performed in the future.

## Supplementary information

**Additional file 1: Table S1**. Allele Frequency from 1000 Genomes Browser and present study.

**Additional file 2: Figure S1**. A: Begg’s funnel plot for publication bias test (A-allele vs. G-allele). Each point represents a separate study for the indicated association. B: Egger’s publication bias plot (A-allele vs. G-allele).

**Additional file 3: Figure S2**. Sensitivity analysis between HTRA1 gene rs11200638 polymorphism and AMD risk (A-allele vs. G-allele).

## Data Availability

All data generated or analyzed during this study are included in this published article and its supplementary information files.

## Abbreviations

AMD: Age-related macular degeneration
GWAS: Genome-wide association studies
HTRA1: High-temperature requirement factor A1
CNV: Choroidal neovascularization
VEGF: Vascular endothelial growth factor
AREDS: Age-Related Eye Disease Study
SNP: Single nucleotide polymorphism
HB: Hospital-based
PB: Population-based
SOC: Source of control
PCR-RFLP: Polymerase chain reaction followed by restriction fragment length polymorphism
MALDI-TOF: A chip-based matrix-assisted laser-desorption/ionization time-of-flight
