# Supplementary material for: HTRA1 rs11200638 variant and AMD risk from a comprehensive analysis about 15,316 subjects
Source: BMC Med Genet. 2020 May 15;21:107. doi: 10.1186/s12881-020-01047-5 (PMC7229611; doi:10.1186/s12881-020-01047-5)
Supplement: Supplementary file 1 — Additional file 1: Table S1. Allele Frequency from 1000 Genomes Browser and present study. [file 12881_2020_1047_MOESM1_ESM.docx]

| Allele Frequency  /Different sources | Global | African | East Asian | Europe | South Asian | American | Case group | Control group |
| --- | --- | --- | --- | --- | --- | --- | --- | --- |
| A-allele % | 0.29 | 0.257 | 0.411 | 0.194 | 0.34 | 0.25 | 0.5422 | 0.365173 |
| G-allele % | 0.71 | 0.743 | 0.589 | 0.806 | 0.66 | 0.75 | 0.457841 | 0.634827 |

Supplementary Table 1 Allele Frequency from 1000 Genomes Browser and present study
