# Supplementary figures and images for: HTRA1 rs11200638 variant and AMD risk from a comprehensive analysis about 15,316 subjects
Source: BMC Med Genet. 2020 May 15;21:107. doi: 10.1186/s12881-020-01047-5 (PMC7229611; doi:10.1186/s12881-020-01047-5)

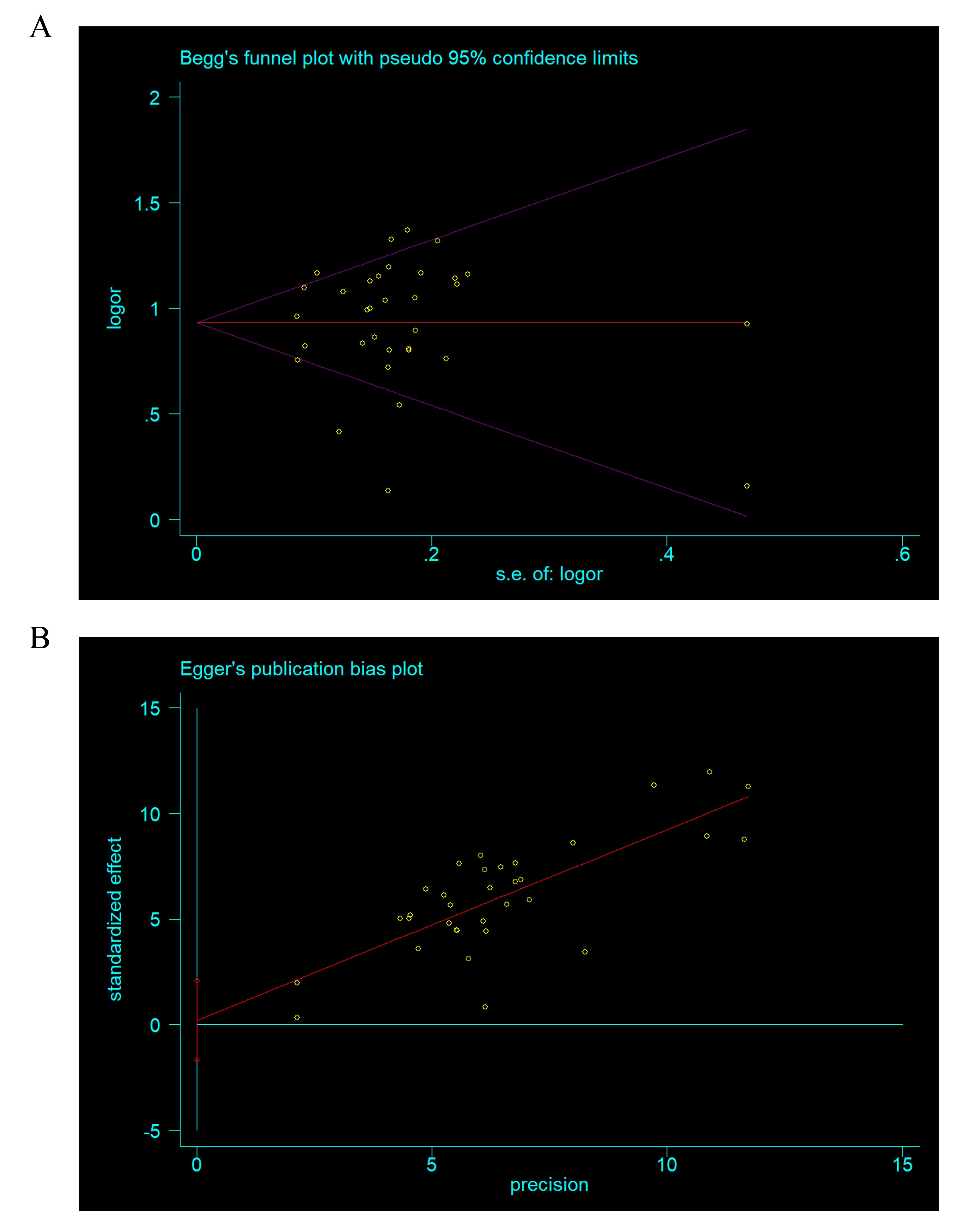

Supplement: Supplementary file 2 — Additional file 2: Figure S1. A: Begg’s funnel plot for publication bias test (A-allele vs. G-allele). Each point represents a separate study for the indicated association. B: Egger’s publication bias plot (A-allele vs. G-allele). [file 12881_2020_1047_MOESM2_ESM.tif]

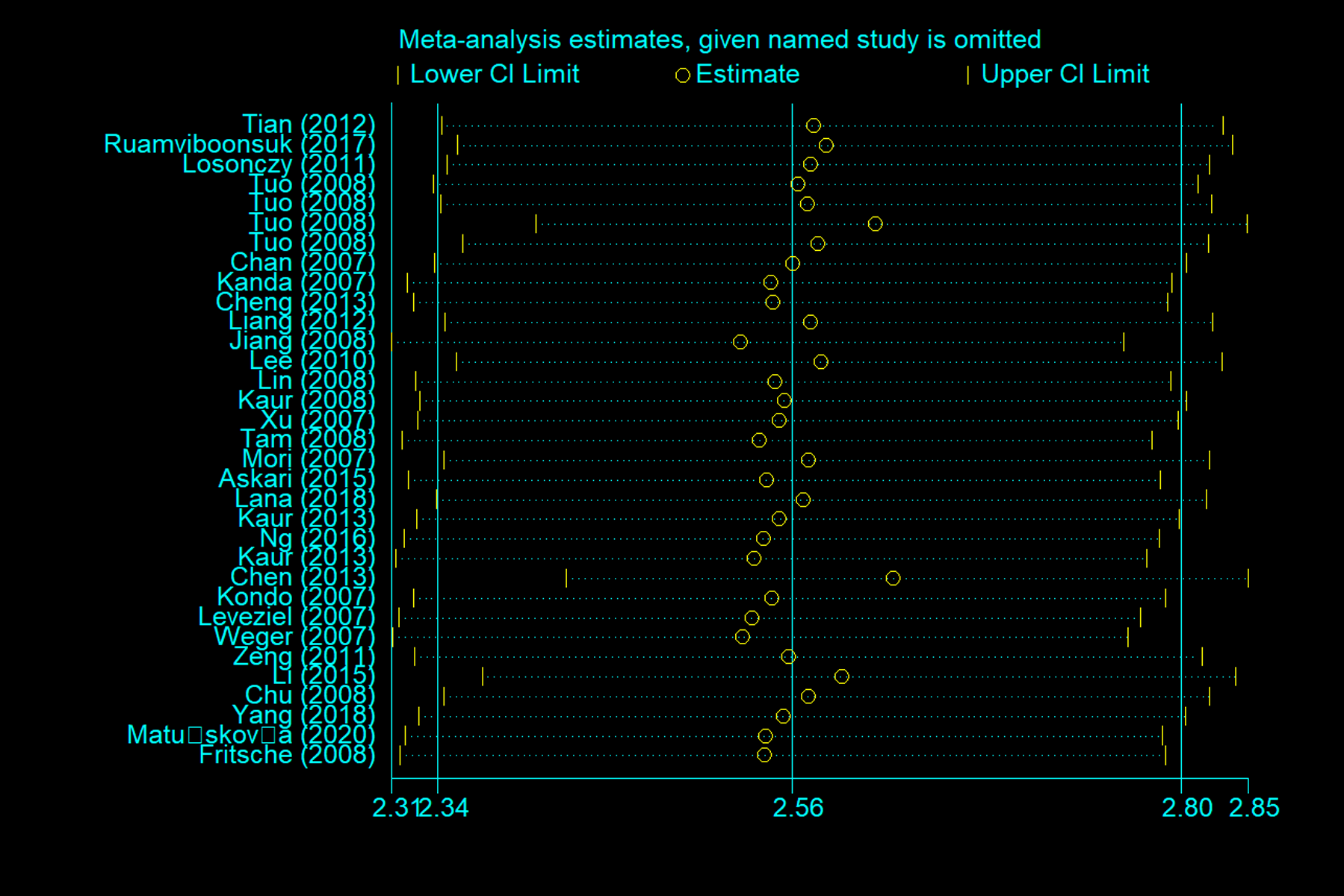

Supplement: Supplementary file 3 — Additional file 3: Figure S2. Sensitivity analysis between HTRA1 gene rs11200638 polymorphism and AMD risk (A-allele vs. G-allele). [file 12881_2020_1047_MOESM3_ESM.tif]
